# Supplementary material for: Inhibition of myeloid-derived suppressor cell arginase-1 production enhances T-cell-based immunotherapy against Cryptococcus neoformans infection
Source: Nat Commun. 2022 Jul 14;13:4074. doi: 10.1038/s41467-022-31723-4 (PMC9283461; doi:10.1038/s41467-022-31723-4)
Supplement: Supplementary file 1 — Supplementary Information [file 41467_2022_31723_MOESM1_ESM.pdf]

SUPPLEMENTARY INFORMATION

Supplementary Figure 1

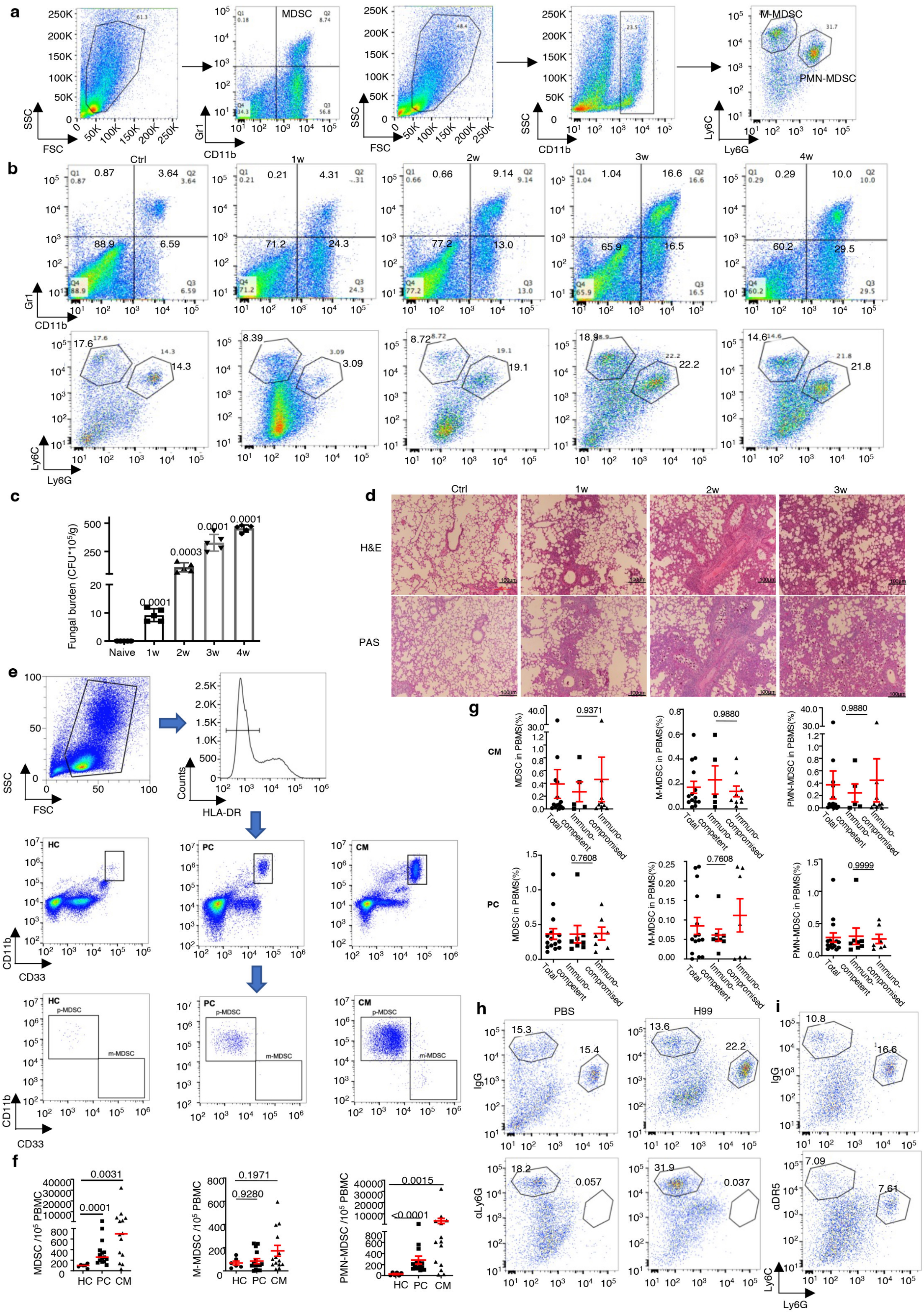

Supplementary Figure 1. ***C. neoformans*** **infection induces the recruitment of MDSCs, especially PMN-MDSCs, to exert deleterious effects. (a)** Gating strategy of $CD11b^+Gr1^+$MDSCs, $CD11b^+Ly6G^+Ly6C^{low}$ M-MDSCs and $CD11b^+Ly6G^-Ly6C^{high}$ PMN-MDSCs among lung cells. **(b)** Representative flow charts of MDSCs, M-MDSCs and PMN-MDSCs in mice without (Ctrl) or with intratracheal infection of *C. neoformans* strain H99 (1×10^3 CFU/mouse) for the indicated weeks (w). **(c-d)** Fungal burden **(c)** and representative histological images with hematoxylin-eosin (H&E) and Periodic Acid-Schiff (PAS) staining **(d)** of the lung in mice at indicated weeks (w) post H99 infection were compared with non-infected group (Ctrl). Scale bars = 100μm. **(e)** Gating strategy and representative charts of human MDSCs ($HLA^-DR^-CD11b^+CD33^+$), M-MDSCs ($HLA\text{-}DR^-CD11b^+CD33^+CD15^-CD14^+$) and PMN-MDSCs ($HLA^-DR^-CD11b^+CD33^+CD15^+CD14^-$) in peripheral blood mononuclear cells (PBMCs) of healthy control (HC), pulmonary cryptococcosis (PC) or cryptococcal meningitis (CM) patients. **(f)** Numbers of MDSCs, M-MDSCs and PMN-MDSCs in 10^5 PBMCs of healthy controls (HC, n=6), and patients with pulmonary cryptococcosis (PC, n=15) or cryptococcal meningitis (CM, n=14) were counted. **(g)** Frequency of MDSC, PMN-MDSCs and M-MDSCs in PBMCs of total, immunocompetent or immunocompromised patients with pulmonary cryptococcosis (PC) and cryptococcal meningitis (CM), respectively. **(h)** Representative flow charts of M-/PMN-MDSCs from H99-infected mice, which were intraperitoneally treated with anti-Ly6G antibody (αLy6G) or control IgG twice a week, **related to Fig 1e. (i)** Representative flow charts of M-/PMN-MDSCs in lungs from mice with intraperitoneal injection of anti-DR5 antibody (αDR5) or control IgG twice a week, **related to Fig 1i.** Data were presented as mean ± SEM, n=5 biologically independent samples (**c**). Data were analyzed by unpaired two-sided Student's t test in **c, f, g**. Source data are provided as a Source Data file.

Supplementary Figure 2

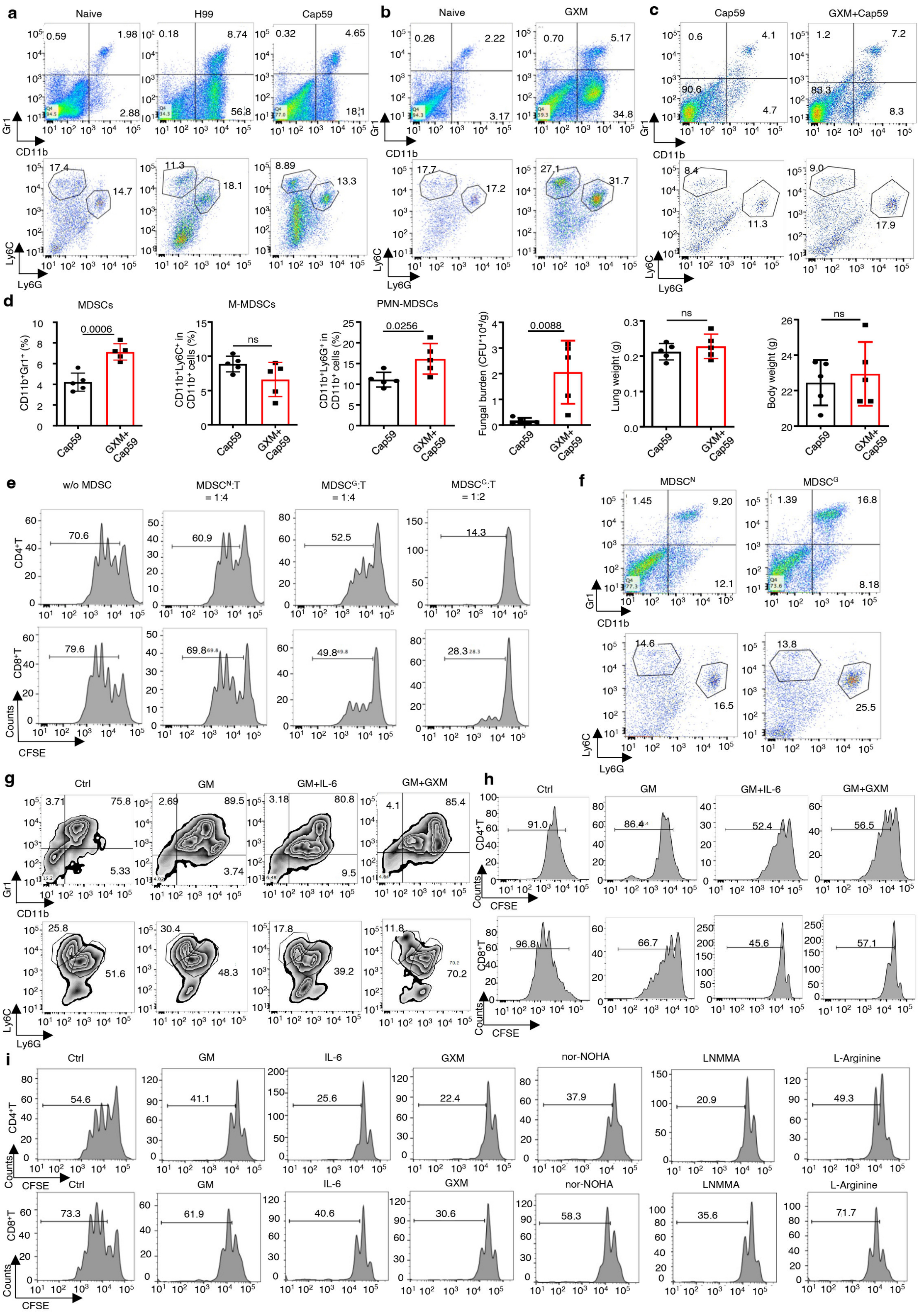

Supplementary Figure 2. GXM induces the expansion and activation of MDSCs, especially PMN-MDSCs, for dampening T-cells and aggravating *C. neoformans* infection. (a) Representative flow charts of MDSCs, M-MDSCs or PMN-MDSCs in mice, which were intratracheally infected with a single exposure of *C. neoformans*

encapsulated strain H99 (1×10^3 CFU/mouse) or hypocapsular strain *cap59Δ* mutant (Cap59, 1×10^5 CFU/mouse) for 14 days, **related to Fig 2a**. **(b)** Representative flow charts of MDSCs, M-MDSCs or PMN-MDSCs in mice treated without or with GXM (100 μg/mouse twice a week) for 14 days, **related to Fig 2c**. **(c, d)** Representative flow charts of MDSCs, M-MDSCs or PMN-MDSCs **(c)** and the percentage of MDSCs, M-MDSCs or PMN-MDSCs, fungal burden, lung weight and body weight **(d)** in mice exposed to *cap59Δ* strain (1×10^5 CFU/mice) without (Cap59) and with pretreatment of GXM (100 μg/mouse twice a week for two weeks) indicated as Cap59+GXM. **(e)** Representative flow charts of $CD4^+$ or $CD8^+$ T cell proliferation, which were co-cultured without (w/o) or with PMN sorted from PBS-treated mice ($MDSC^N$) or MDSCs sorted from GXM-treated mice ($MDSC^G$), **related to Fig 2e**. **(f)** Representative flow charts of MDSCs, M-MDSCs, PMN-MDSCs of Cap59-infected wild-type mice (WT) for 14 days, which are adoptively transferred with $MDSC^N$ or $MDSC^G$, **related to Fig 2g**. **(g)** Representative flow charts of bone marrow (BM)-derived MDSCs, M-MDSCs and PMN-MDSCs, which were induced by medium (Ctrl), GM-CSF (GM, 40 ng/ml), GM-CSF+IL-6 (40 ng/ml) or GM-CSF+GXM (10 μg/well) for 5 days, **related to Fig 2j**. **(h)** Representative results of $CD4^+$ or $CD8^+$ T cell proliferation ratio, which were co-cultured for 72h without (w/o) or with BM-derived MDSCs generated as described in (**g**), **related to Fig 2l**. **(i)** Examples of flow charts showing proliferation frequency of $CD4^+$ or $CD8^+$ T cells, which were pretreated with nitric oxide synthase inhibitor L-NMMA, arginase inhibitor nor-NOHA, or L-arginine and then co-cultured for 72h with BM-MDSCs generated by GM + GXM as described in (**g**), **related to Fig 2m**. Data were presented as mean ± SEM, n=5(d) biologically independent samples. Data were analyzed by unpaired two-sided Student's t test. Source data are provided as a Source Data file.

Supplementary Figure 3

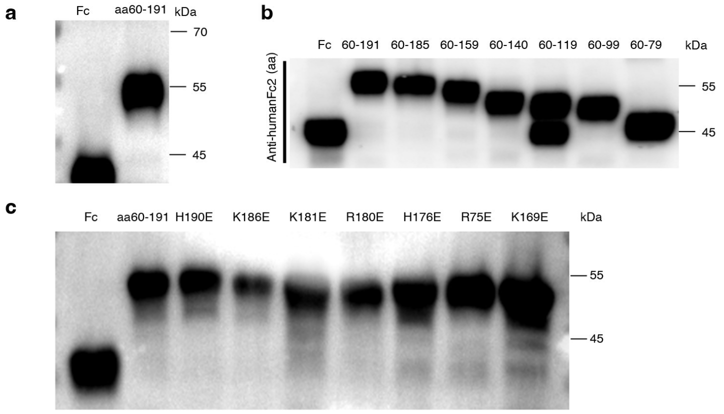

Supplementary Figure 3. CLEC2D specifically recognizes GXM from *C. neoformans*.

(a-c) Representative blots of expression verification for full length **(a)**, truncated **(b)** or mutant **(c)** human CLEC2DCTLD (C-type lectin-like domain) in fusion with human IgG1-Fc peptides by anti-humanFc. Representative blots were shown by three independent experiments. Source data are provided as a Source Data file.

Supplementary Figure 4

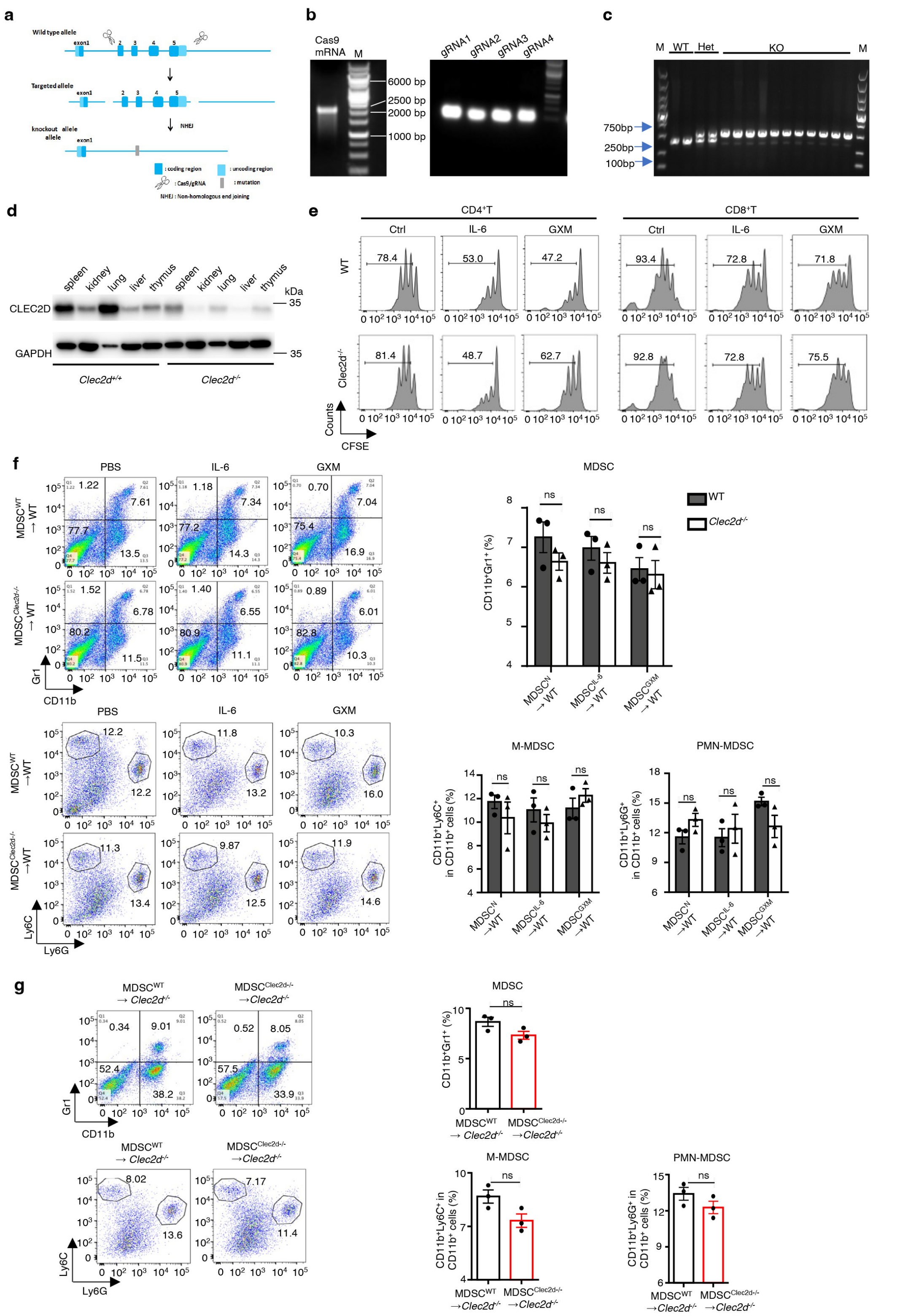

Supplementary Figure 4. ***Clec2d*** **deficiency impairs MDSC activation to alleviate C. neoformans infection. (a)** The design strategy of constructing *Clec2d* knockout mice. **(b)** Eletrophoresis images of *Cas9* mRNA and gRNAs obtained by *in vitro* transcription. **(c)** Agarose gel images showing PCR products of genome of WT,

heterozygous (Het) and homozygous (KO) *Clec2d* mutant mice. *Clec2d*$^{-/-}$ mice were verified by PCR before each experiment and representative gels were shown. (**d**) Plots showing *Clec2d* expression in different organs of WT or *Clec2d*-deficient mice with GAPDH as a loading control. Representative blots of three independent experiments were shown. **(e)** Representative flow charts showing proliferation frequency of CD4^{+} or CD8^{+} T cells cocultured for 72h with wild-type or *Clec2d*-deficient BM-derived MDSCs, which were generated with GM-CSF (GM, 40 ng/ml, MDSC^{N}), GM+IL-6 (40 ng/ml, MDSC^{IL-6}) or GM+GXM (10 μg/well, MDSC^{GXM}) for 5 days, related to Fig 4e. (**f**) The percentage of MDSCs, M-/PMN-MDSCs in lungs of Cap59-infected mice (1×10^{3} CFU/mouse), which are adoptively transferred with MDSC^{N}, MDSC^{IL-6} or MDSC^{GXM}. Representative flow charts were shown in the left panel. **(g)** The frequency of MDSCs, M-/PMN-MDSCs in lungs of H99-infected *Clec2d*$^{-/-}$ mice (1×10^{3} CFU/mouse), which are adoptively transferred with MDSC^{WT} and MDSC$^{Clec2d-/-}$ (1×10^{6} cells/mouse) generated by GM (40 ng/ml) + GXM (10 μg/well). Representative flow charts were shown in the left panel. Data were presented as mean ± SEM, n=3 biologically independent samples (**f, g**). Data were analyzed by unpaired two-sided Student's t test in **f, g**. Source data are provided as a Source Data file.

Supplementary Figure 5

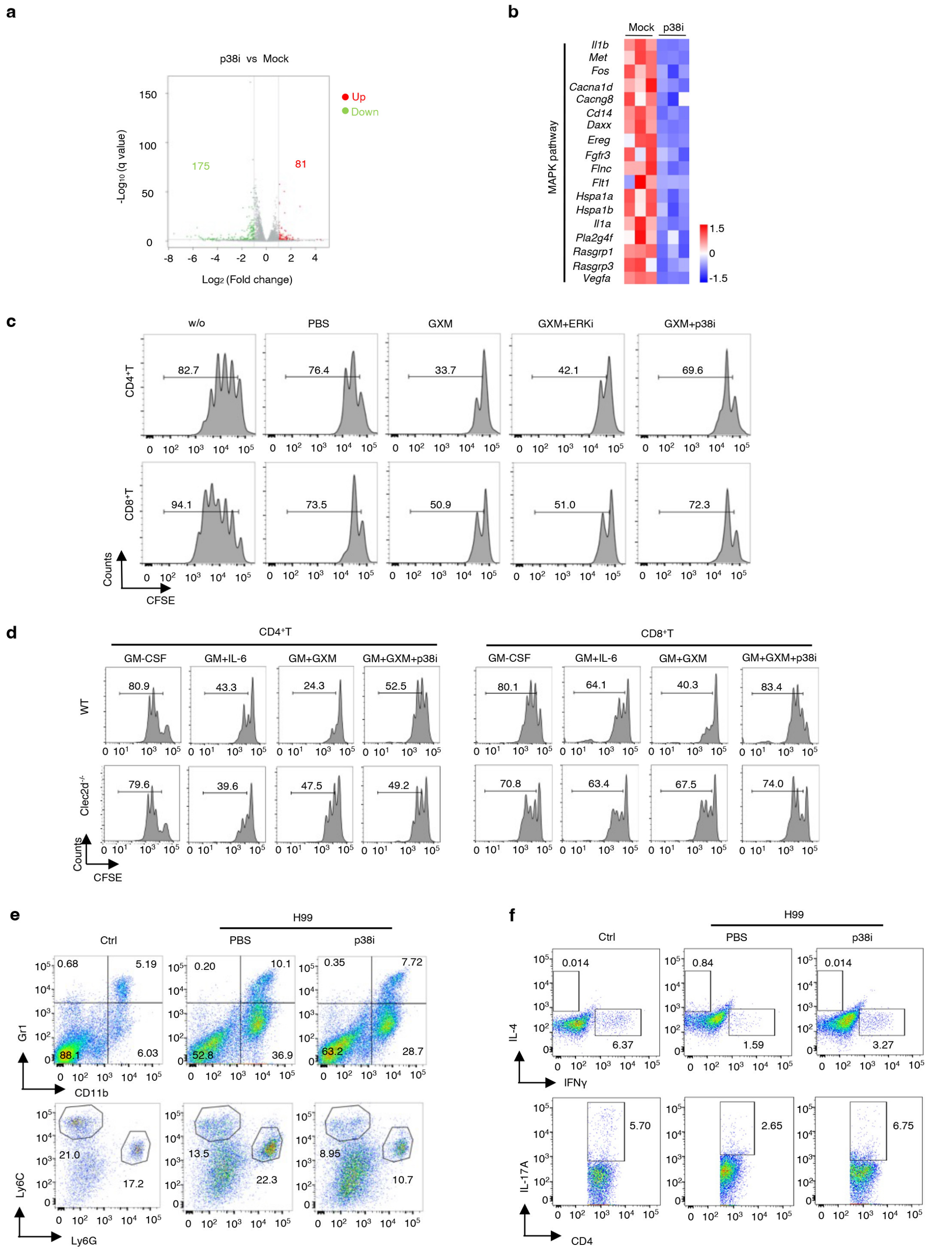

Supplementary Figure 5. Inhibiting p38 activation is an effective immunotherapy against *C. neoformans* infection. **(a)** 175 downregulated (green) and 81 upregulated genes (red) in BM-derived MDSCs from wild-type mice, which were generated by GM-CSF (40 ng/ml) + GXM (10 µg/well) for 5 days and then treated with GXM (10 µg/well) and SB202109 (p38i, 10 µM) for 3h were shown in Volcano plot, with differential expression based on absolute fold change >2 and FDR < 0.05. **(b)** Heatmap showing

the expression of genes involved in MAPK signaling pathway of Mock and p38i group, indicated in **(a)**. **(c)** Representative flow charts showing proliferation of CD4$^+$ or CD8$^+$ T cells cocultured with MSC-2, which were untreated (w/o) or treated with PBS, GXM (10 μg/well), GXM+ SB202190 (GXM + p38i, 10 μM) or GXM + U0126 (GXM + Erki, 10 μM) for 6h, **related to Fig 5f**. **(d)** Representative flow charts of proliferation of CD4$^+$ and CD8$^+$ T cells co-cultured with wild-type or *Clec2d*$^{-/-}$ BM-derived MDSCs, which were generated with GM-CSF (40 ng/ml), GM-CSF+IL-6 (GM+IL-6, 40 ng/ml), GM-CSF+GXM (GM+GXM, 10 μg/well) or GM-CSF+GXM+SB202190 (GM+GXM+p38i, 10 μM) for 5 days, **related to Fig 5g**. **(e, f)** Representative flow charts of MDSCs, M-MDSCs, PMN-MDSCs **(e)**, and IFNγ$^+$ Th1 and IL-17A$^+$ Th17 **(f)** in the lungs of H99-infected mice (1×10^3 CFU/mouse) on Day 14, which were intraperitoneally treated without (PBS) or with p38 inhibitor SB202190 (p38i, 25 μg/kg every other day), **related to Fig 5k, 5m respectively.** Source data are provided as a Source Data file.

Supplementary Figure 6

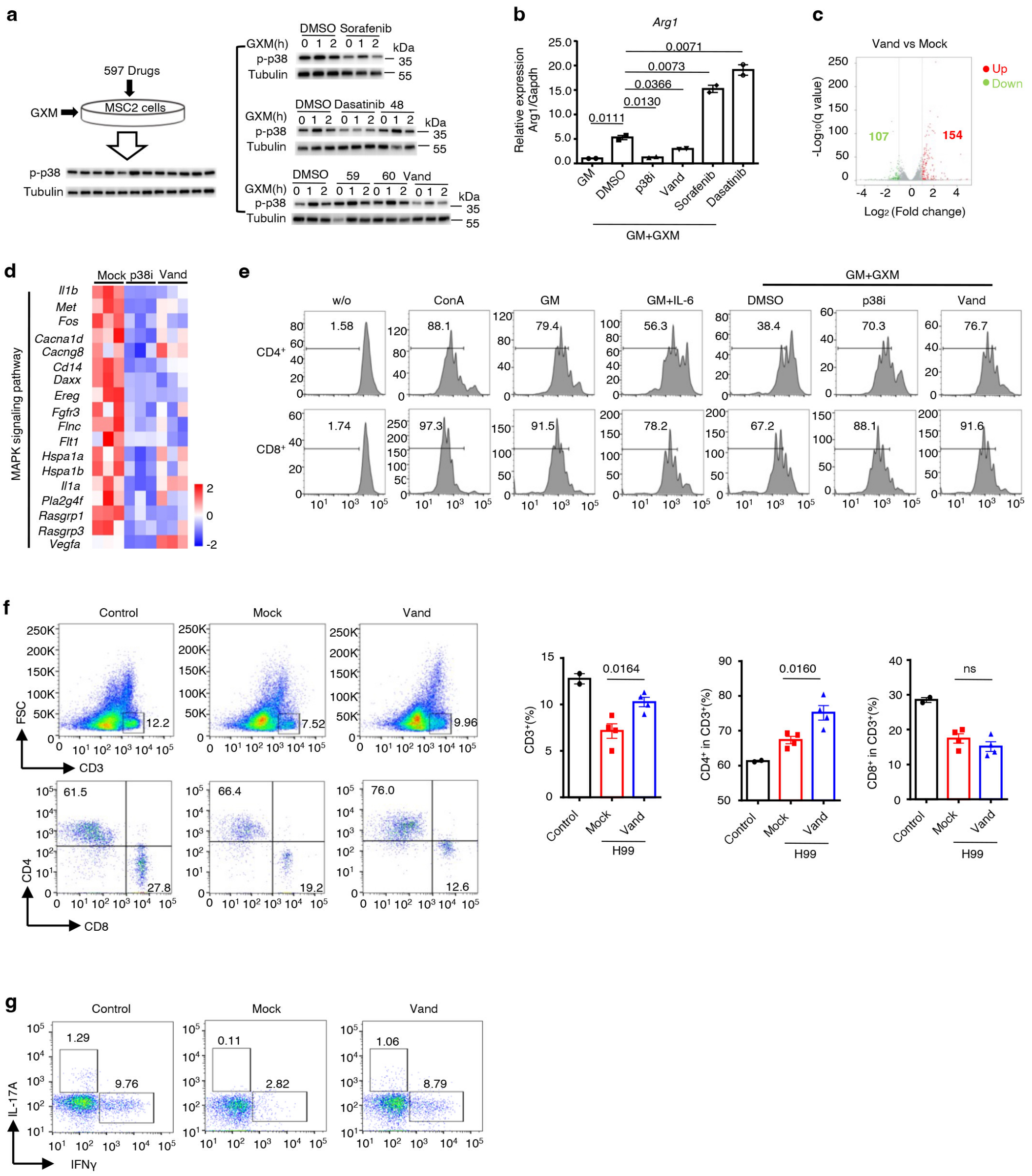

Supplementary Figure 6. Vandetanib treatment restrains cryptococcal infection by impairing GXM-induced p38 activation and Arg1 production in MDSC. (a-b) Screening for drugs which block MDSC suppressive activity. **(a)** Schematic graph showed that 597 drugs were screened for inhibiting GXM-induced p38 phosphorylation with MSC2 cell line and sorafinib, dasatinib and vandetanib were found. Representative blots were shown for three independent experiments. **(b)** Relative mRNA level of *Arg1* was measured in wild-type BM-derived MDSCs, which were generated by GM-CSF (40 ng/ml), GM-CSF+GXM (GM+GXM, 10 μg/well), GM-CSF+GXM+SB202190 (p38i, 10 μM), GM-CSF+GXM+vandetanib (Vand, 2 μM), GM-CSF+GXM+ sorafenib

(sorafenib, 2 μM), GM-CSF+GXM+ dasatinib (dasatinib, 2 μM) for 5 days. **(c)** 154 up-regulated (red) and 107 down-regulated (green) genes in vandetanib-treated (Vand, 5 μM) BM-derived MDSCs, which were generated by GM-CSF (40 ng/ml) + GXM (10 μg/well) for 5 days and then treated with GXM (10 μg/well) for 3h were shown in volcano plot. And differentially expressed genes were identified based on absolute fold change >2 and FDR < 0.05. **(d)** Heatmap showing expression of MAPK signaling pathway-involved genes in wild-type BM-derived MDSCs, which were generated by GM-CSF (GM, 40 ng/ml) + GXM (10 μg/well) and then treated with GXM (10 μg/well) combined with p38 inhibitor SB202190 (p38i, 10 μM) or vandetanib (Vand, 5 μM) for 3h. **(e)** Representative histograms of proliferation frequency of $CD4^+$ and $CD8^+$ T cells, co-cultured with wild-type BM-derived MDSCs, which were generated with GM-CSF (GM, 40 ng/ml), GM-CSF+IL-6 (GM+IL-6, 40 ng/ml), GM-CSF+GXM (GM+GXM, 10 μg/well), GM-CSF+GXM+SB202190 (p38i, 10 μM) or GM-CSF+GXM+vandetanib (Vand, 2 μM) for 5 days, **related to Fig 6e**. **(f)** Percentage of $CD3^+$ T cell, $CD4^+$ and $CD8^+$ in $CD3^+$ T cell of lung in H99-infected mice (1×10^3 CFU/mouse), which were intraperitoneally treated without (Mock) or with vandetanib (Vand, 50 mg/kg every other day). Representative charts were shown in the left panel. **(g)** Representative graphs of pulmonary $IFN\gamma^+$ T_H1 and IL-$17A^+$ T_H17 cells in H99-infected mice (1×10^3 CFU/mouse) without (Mock) or with treatment of vandetanib (Vand, 50 mg/kg every other day), **related to Fig 6j**. Data were presented as mean ± SEM, n=3 (**b**), n=4 (**f**) biologically independent samples. Data were analyzed by unpaired two-sided Student's t test in **b, f**. Source data are provided as a Source Data file.

Supplementary Table 1 Characteristics of patients in Pulmonary Cryptococcosis and Cryptococcal Meningitis study groups

Parameter		Healthy Control	Pulmonary Cryptococcosis*	Cryptococcal Meningitis
n		6	15	14
Sex	Male (n)	2	10	8
	Female (n)	4	5	6
Age in years (mean, range)		28.0, 24-33	55.0, 39-59	58.0, 48-70
Latex agglutination test		-	1, 0-80	20480, 2560-40960
Immune cell types (mean±SEM)				
	WBC (×10^9/L)	5.3±0.5	5.6±2.1	9.0±3.9
	Lymphocytes %	24.8±7.1	25.4±8.1	14.4±8.1
	Neutrophils %	58.2±8.2	62.7±12.8	72.5±22.3
	Eosinophils %	1.7±0.5	1.7±1.3	1.0±0.9
	Monocyte %	8.3±3.9	8.6±4.8	6.4±2.2
Underlying conditions†			46.7% (7/15)	64.3% (9/14)
	Auto immune disease	-	26.7% (4/15)	28.6% (4/14)
	Type II diabetes mellitus	-	20.0% (3/15)	21.4% (3/14)
	Administration of glucocorticoids or immunosuppressants	-	13.3% (2/15)	21.4% (3/14)
	Renal insufficiency	-	6.7% (1/15)	14.3% (2/14)
	Liver cirrhosis		13.3% (2/15)	-
	Solid organ tumor	-	-	7.1% (1/14)
	Head trauma history	-	-	7.1% (1/14)
	Hypophysis	-	-	7.1% (1/14)

*Six of 15 cases were diagnosed by pathological examination after operation, and the serum latex agglutination tests were negative.

†Two and five of pulmonary cryptococcosis and cryptococcal meningitis cases had more than 1 underlying conditions, respectively.

Supplementary Table 2 Primers used in this study

**Primer sequences for Clec2d knockout mice detection**	
P1 F	TAACTCCTGTCCTCTAG
P2 R	ATATGGGAGCCACAACTG
P3 R	GAGAACACCAATCTCAAAAAAC
**Primer sequences for qPCR**	
Mouse Arg-1 F	AAGAATGGAAGAGTCAGTGTGG
Mouse Arg-1 R	GGGAGTGTTGATGTCAGTGTG
Mouse inos F	GAACTGTAGCACAGCACAGGAAAT
Mouse inos R	CGTACCGGATGAGCTGTGAAT
Mouse Clec2d F	GGTTTGACAACCAGGATGAGC
Mouse Clec2d R	TCTCCCCGGATGGGAATCG
Mouse Cd69 F	AGGCTTGTACGAGAAGTTGGA
Mouse Cd69 R	AGTTCACCAGAATATCGCTTCAG
Mouse Mincle F	AGTGCTCTCCTGGACGATAG
Mouse Mincle R	CCTGATGCCTCACTGTAGCAG
Mouse Gapdh F	TGGCCTTCCGTGTTCCTAC
Mouse Gapdh R	GAGTTGCTGTTGAAGTCGCA
Human Clec2d F	AGTCTTGCACTTGTCACGAATTCGAGAGCTAACTGCCATCAAGA
Human Clec2d R	GCATGTGTGAGTTTTGTCAGATCTGACATGTATATCTGATTTGG
**Primer sequences for Clec2d knockout mice generation**	
sgRNA 1	GTCCTAGCCAGTCTATTCTTAGG
sgRNA 2	TCCTAGCCAGTCTATTCTTAGGG
sgRNA 3	GATCAGCAGTGCCAATAAAAGGG
sgRNA 4	GCTTACTGTGAGTGGTGATGTGG

Supplementary Table 3 Regents used in this study

REAGENT or RESOURCE	SOURCE	IDENTIFIER
**Antibodys**		
**For Flow cytometry**		
PerCP-Cy5.5 -anti-mouse CD11b (M1/70)	BD Pharmingen	561114
APC-anti-mouse Gr1(RB6-8C5)	BD Pharmingen	553129
BV421-anti-mouse Ly-6G (1A8)	Biolegend	127628
FITC-anti-mouse Ly-6G (1A8)	eBioscience	11-9668-82
PE- anti-mouse Ly6C (HK1.4)	Biolegend	128008
APC-anti-mouse CD8 (53-6.7)	Biolegend	100712
BV421-anti-mouse CD4 (GK1.5)	Biolegend	100438
APC-anti-mouse IFNγ (XMG1.2)	Biolegend	505810
PE-anti-mouse IL-17A (ebio17B7)	Biolegend	559502
Alexa Fluor 594- anti-mouse IgG	abcam	ab150116
FITC-anti-human Fc fragment	Jackson Immuno Research	109-007-008
**For western blot**		
phospho-p38	Cell Signaling Technology	4511S
p38	Cell Signaling Technology	8690S
phospho-ERK	Cell Signaling Technology	4370S
ERK	Cell Signaling Technology	4695S
GAPDH	Cell Signaling Technology	5174S
phospho-p38	Cell Signaling Technology	4511S
p38	Cell Signaling Technology	8690S
**For Neutralization**		
Anti-mouse Ly6G (1A8)	BioX Cell	BE0075
Anti-mouse DR5 (MD5-1)	BioX Cell	BE0161
Rat IgG1 isotype control (TNP6A7)	BioX Cell	BP0290
**inhibitors**		
SB202190	MCE	HY-10295
U0126-Etoh	Selleck	S1102
**Experimental Models: Organisms/Strains**		
C57BL/6 wild-type mice	Charles River, China	213
Clec2d$^{-/-}$ mice	This study	N/A
C. neoformans serotype A (H99)	Jia's lab	N/A
C. neoformans serotype A mutation (Cap59)	Jia's lab	N/A
**Primers**		
See Table S1	This study	Table S1
Recombinant DNA		
Plasmid: pFuse-hIgG1-Fc	InvivoGen	pFuse-hIgG1-Fc
**Chemicals, Peptides, and Recombinant**		

**Proteins**		
Collagenase type II	Serva	17454
Dulbecco's modified Eagle's medium and Nutrient Mixture F-12	Thermo Fisher Scientific	11320-082
Fetal bovine serum	Thermo Fisher Scientific	10091148
Penicillin/streptomycin	Thermo Fisher Scientific	15140-122
Pentobarbital sodium salt	Merk	1063180500
Gentamicin	MCE	HY-A0276
Triton X-100	Sigma	X100
0.05% Trypsin-EDTA	Thermo Fisher Scientific	25300062
Lipofectamine® 3000 Transfection Reagent	Thermo Fisher Scientific	L3000008
Dulbecco's Modified Eagle's Medium	Thermo Fisher Scientific	11965-092
dialysis membranes 7000D	Solarbio	YA1082-5M
polyethylene glycol-2000	Sangon Biotech	A601785-0500
Opti-MEM	Thermo Fisher Scientific	31985070
Lipofectamine® RNAiMAX Transfection Reagent	Thermo Fisher Scientific	13778075
TRIzol	Thermo Fisher Scientific	10296010
Cell lysis buffer for Western	Beyotime	P0013J
RIPA buffer	Beyotim	P0013C
**Critical Commercial Assays**		
RNeasy Micro Kit	Qiagen	74004
Fast Site-Directed Mutagenesis Kit	TIANGEN	KM101
FastQuant RT Kit (With gDNase)	TIANGEN	KR106-02
SuperReal PreMix Plus (SYBR Green)	TIANGEN	FP205-02
LEGEND MAX™ Mouse GM-CSF ELISA Kit	Biolegend	439807
**Software and Algorithms**		
Feature Extraction software version10.7.1.1	Agilent	N/A
Genespring version13.1	Agilent	N/A
gel imaging system Tanon 5500	Tanon	
GraphPad prism 8	GraphPad	http://www.graphpad.com RRID:SCR_002798
FlowJo software version V10	Tree Star	N/A
