# Supplementary material for: Inhibition of myeloid-derived suppressor cell arginase-1 production enhances T-cell-based immunotherapy against Cryptococcus neoformans infection
Source: Nat Commun. 2022 Jul 14;13:4074. doi: 10.1038/s41467-022-31723-4 (PMC9283461; doi:10.1038/s41467-022-31723-4)
Supplement: Supplementary file 3 — Reporting Summary [file 41467_2022_31723_MOESM3_ESM.pdf]

nature portfolio

Corresponding author(s): Xin-Ming Jia and Jie-Ming Qu

Last updated by author(s): May 19, 2022

Reporting Summary

Nature Portfolio wishes to improve the reproducibility of the work that we publish. This form provides structure for consistency and transparency in reporting. For further information on Nature Portfolio policies, see our Editorial Policies and the Editorial Policy Checklist.

Statistics

For all statistical analyses, confirm that the following items are present in the figure legend, table legend, main text, or Methods section.

n/a	Confirmed	
☐	☒	The exact sample size (n) for each experimental group/condition, given as a discrete number and unit of measurement
☐	☒	A statement on whether measurements were taken from distinct samples or whether the same sample was measured repeatedly
☐	☒	The statistical test(s) used AND whether they are one- or two-sided<br>*Only common tests should be described solely by name; describe more complex techniques in the Methods section.*
☐	☒	A description of all covariates tested
☐	☒	A description of any assumptions or corrections, such as tests of normality and adjustment for multiple comparisons
☐	☒	A full description of the statistical parameters including central tendency (e.g. means) or other basic estimates (e.g. regression coefficient) AND variation (e.g. standard deviation) or associated estimates of uncertainty (e.g. confidence intervals)
☐	☒	For null hypothesis testing, the test statistic (e.g. F, t, r) with confidence intervals, effect sizes, degrees of freedom and P value noted<br>*Give P values as exact values whenever suitable.*
☒	☐	For Bayesian analysis, information on the choice of priors and Markov chain Monte Carlo settings
☒	☐	For hierarchical and complex designs, identification of the appropriate level for tests and full reporting of outcomes
☒	☐	Estimates of effect sizes (e.g. Cohen's d, Pearson's r), indicating how they were calculated

Our web collection on statistics for biologists contains articles on many of the points above.

Software and code

Policy information about availability of computer code

Data collection	Flow cytometry data was collected by FACSVerse (BD Biosciences) using BD DIVA8.0.1 software (BD Biosciences).<br>Flow sorting was performed by FACSAria II (BD Biosciences) .<br>qPCR was done by Roche LightCycler96 sequencing detection system.<br>RNA integrity was evaluated using the Agilent 2100 Bioanalyzer (Agilent Technologies, Santa Clara, CA, USA). The libraries were sequenced on the Illumina sequencing platform (HiSeqTM 2500 or Illumina HiSeq X Ten)
Data analysis	Flow cytometry data was analyzed by Flowjo v10.<br>Data analysis and plotted were using GraphPad Prism 8.

For manuscripts utilizing custom algorithms or software that are central to the research but not yet described in published literature, software must be made available to editors and reviewers. We strongly encourage code deposition in a community repository (e.g. GitHub). See the Nature Portfolio guidelines for submitting code & software for further information.

Data

Policy information about availability of data

All manuscripts must include a data availability statement. This statement should provide the following information, where applicable:

- Accession codes, unique identifiers, or web links for publicly available datasets
- A description of any restrictions on data availability
- For clinical datasets or third party data, please ensure that the statement adheres to our policy

The RNA-seq raw and preprocessed data in this study have been deposited in the SRA database under accession code PRJNA799667, https://www.ncbi.nlm.nih.gov/sra/PRJNA799667. The data supporting the findings from this study are available within the article file and its supplementary information/Source Data file. Any

other raw data or non-commercial material used in this study are available from the corresponding author upon reasonable request. Source data are provided with this paper.

Field-specific reporting

Please select the one below that is the best fit for your research. If you are not sure, read the appropriate sections before making your selection.

☒ Life sciences ☐ Behavioural & social sciences ☐ Ecological, evolutionary & environmental sciences

For a reference copy of the document with all sections, see nature.com/documents/nr-reporting-summary-flat.pdf

Life sciences study design

All studies must disclose on these points even when the disclosure is negative.

Sample size	The minimum of samples in each experiment was n=3, and up to n=10. The exact n for each experiment was described in corresponding figure legends. Sample sizes were determined based on previous experience and standards in the field. Low variability between the same type of samples, indicated as SEM, confirming that n=3-5 samples is sufficient to observe statistically significant differences between relevant groups. whereas for survival rate experiments, at least n=8 samples were used as figure legend indicated.
Data exclusions	No data was excluded from analysis
Replication	At least three independent experiments were performed for each experiments, each panel presented the representative data. All reported data were reproduced reliably.
Randomization	For animal models, all mice (WT or Clec2d-/-) used in this study were divided into different treatment groups randomly. For cell culture, like MDSC in vitro generation, T-cell proliferation assays, or MDSCs for real-time PCR, cells were divided into each plates and assigned into different treatments groups randomly.
Blinding	Human studies were single-blinded design, and all data acquisition and analysis in this study were performed in a blinded way.

Reporting for specific materials, systems and methods

We require information from authors about some types of materials, experimental systems and methods used in many studies. Here, indicate whether each material, system or method listed is relevant to your study. If you are not sure if a list item applies to your research, read the appropriate section before selecting a response.

Materials & experimental systems

n/a	Involved in the study	
☐	☒	Antibodies
☐	☒	Eukaryotic cell lines
☒	☐	Palaeontology and archaeology
☐	☒	Animals and other organisms
☐	☒	Human research participants
☒	☐	Clinical data
☒	☐	Dual use research of concern

Methods

n/a	Involved in the study	
☒	☐	ChIP-seq
☐	☒	Flow cytometry
☒	☐	MRI-based neuroimaging

Antibodies

Antibodies used	Antibodies used in this paper:<br>For Flow cytometry<br>PerCP-Cy5.5 -anti-mouse CD11b (M1/70) BD Pharmingen 561114; 1: 100<br>APC-anti-mouse Gr1(RB6-8C5) BD Pharmingen 553129; 1:100<br>BV421-anti-mouse Ly-6G (1A8) Biolegend 127628; 1:100<br>FITC-anti-mouse Ly-6G (1A8) eBioscience 11-9668-82; 1:100<br>PE- anti-mouse Ly6C (HK1.4) Biolegend 128008; 1:100<br>APC-anti-mouse CD8 (53-6.7) Biolegend 100712; 1:100<br>BV421-anti-mouse CD4 (GK1.5) Biolegend 100438; 1:100<br>APC-anti-mouse IFNγ (XMG1.2) Biolegend 505810; 1:100<br>PE-anti-mouse IL-17A (ebio17B7) Biolegend 559502; 1:100<br>For microscopy<br>Alexa Fluor 594-labeled donkey anti-mouse IgG abcam ab150116; 1:200<br>FITC-anti-human Fc fragment Jackson Immuno Research 109-007-008; 1:50<br>For western blot<br>phospho-p38 Cell Signaling Technology 4511S; 1:1000<br>p38 Cell Signaling Technology 8690S; 1:1000

phospho-ERK Cell Signaling Technology 4370S; 1:1000
ERK Cell Signaling Technology 4695S; 1:1000
GAPDH Cell Signaling Technology 5174S; 1:1000
CLEC2D monoclonal antibody was generated by our lab (Clone 2260CT10.1.3.2).

For Neutralization
Anti-mouse Ly6G (1A8) BioX Cell BE0075; 200mg/mice once
Anti-mouse DR5 (MD5-1) BioX Cell BE0161; 200mg/mice once
Rat IgG1 isotype control (TNP6A7) BioX Cell BP0290; 200mg/mice once

Validation

All antibodies were validated before first usage. This involve appropriate negative and positive controls.

For Flow cytometry antibodies, we relied on the manufacturer's validation which were used in many labs and published papers, and we performed isotype control when we first use, including PerCP-Cy5.5 -anti-mouse CD11b (M1/70), APC-anti-mouse Gr1(RB6-8C5), BV421-anti-mouse Ly-6G (1A8), FITC-anti-mouse Ly-6G (1A8) eBioscience, PE- anti-mouse Ly6C (HK1.4) Biolegend, APC-anti-mouse CD8 (53-6.7) Biolegend , BV421-anti-mouse CD4 (GK1.5) Biolegend , APC-anti-mouse IFNγ (XMG1.2) Biolegend , PE-anti-mouse IL-17A (ebio17B7) Biolegend.

For microscopy
Detailed validation from manufacturer's data sheets:
-Alexa Fluor 594-labeled donkey anti-mouse IgG abcam ab150116
IHC-Fr, ICC/IF were validated in published papers, the recommended concentration is 1/200-1/1000
-FITC-anti-human Fc fragment Jackson Immuno Research 109-007-008
Product was used in published papers, the recommended concentration is 20-40μg/ml

For western blot
Detailed validation from manufacturer's data sheets:
-phospho-p38 Cell Signaling Technology 4511S;
Image was western blot analysis of extracts from COS and 293 cells, untreated or UV-treated, using Phospho-p38 MAPK (Thr180/Tyr182) (D3F9) XP® Rabbit mAb
-p38 Cell Signaling Technology 8690S;
Image was western blot analysis of extracts from various cell lines using p38 MAPK (D13E1) XP® Rabbit mAb.
-phospho-ERK Cell Signaling Technology 4370S;
Western blot analysis of extracts from 293, NIH/3T3 and C6 cells, treated with λ phosphatase or TPA #4174 as indicated, using Phospho-p44/42 MAPK (Erk1/2) (Thr202/Tyr204) (D13.14.4E) XP® Rabbit mAb (4370s).
-ERK Cell Signaling Technology 4695S;
Western blot analysis of extracts from HeLa, NIH/3T3 and C6 cells, using p44/42 MAPK (Erk1/2) (137F5) Rabbit mAb.
-GAPDH Cell Signaling Technology 5174S;
Western blot analysis of extracts from various cell lines using GAPDH (D16H11) XP® Rabbit mAb.
- CLEC2D monoclonal antibody (Clone 2260CT10.1.3.2).
Western blot analysis of extracts from primary bone-marrow cells, and Raw264.7 serves as a negative control in which CLEC2D was not expressed.

For Neutralization
These two neutralizing antibodies, nti-mouse Ly6G (1A8) BioX Cell BE0075 and anti-mouse DR5 (MD5-1) BioX Cell BE0161 were widely used in many labs and validated by published papers. We also validated their neutralizing function by using isotype rat IgG1 isotype control (TNP6A7) BioX Cell BP0290, and Flow cytometry results showed Ly6G+ MDSCs were depleted in figure 1e and 1i.

Eukaryotic cell lines

Policy information about cell lines

Cell line source(s)	The immortalized MDSC cell line MSC-2 was a gift from Professor Zhihai Qin at the Institute of Biophysics, Chinese Academy of Sciences.
Authentication	This cell line was passaged in laboratory and authenticate by sinobiological company.
Mycoplasma contamination	MSC-2 cell line was tested regularly by using GMyc-PCR Mycoplasma Test Kit (40601ES10, Yeasen, China) and has no mycoplasma contamination.
Commonly misidentified lines (See ICLAC register)	No commonly misidentified cell lines were included in this study.

Animals and other organisms

Policy information about studies involving animals; ARRIVE guidelines recommended for reporting animal research

Laboratory animals	C57BL/6 mice were purchased from Vital River (Shanghai, China). The Clec2d knockout mouse line was generated using CRISPR–Cas9 methods. All animal studies were performed with sex- (female) and age-matched (6-8weeks) mice. All mice were bred under specific pathogen-free conditions in ventilated cages with ad libitum food and water at Tongji University School of Medicine (Shanghai, China). Housing conditions were as following: dark/light cycle 12/12, ambient temperature around 21–22 °C and humidity between 40 and 70%.
Wild animals	No wild animals were involved in this study.
Field-collected samples	This study did not involve field-collected samples
Ethics oversight	All animal studies were performed with the approval from the Institutional Laboratory Animal Care and Use Committee of Tongji University School of Medicine (Shanghai, China) (protocol No. TJAA09021101).

Note that full information on the approval of the study protocol must also be provided in the manuscript.

Human research participants

Policy information about studies involving human research participants

Population characteristics	Human subjects contain healthy controls (n=6) or patients with pulmonary cryptococcosis (n=15) or cryptococcal meningitis (n=14) was collected at Shanghai Huashan Hospital, Fudan University School of Medicine (Shanghai, China). The diagnosis of pulmonary cryptococcosis or cryptococcal meningitis was based on a combination of clinical and radiological suspicion and laboratory . And the characteristics of patients in each study group are shown in Supplementary Table S1.
Recruitment	Participants were recruited via advertising on hospital web-pages and suggested by clinical doctors.
Ethics oversight	Studies of human PBMCs were approved by the Human Research Committee of Shanhai Jiaotong University School of Medicine (protocol No. KY2020-334). All participants involved in this study were informed orally and in writing of objectives, contents, risks, and discomforts associated with participation before writing informed consent.

Note that full information on the approval of the study protocol must also be provided in the manuscript.

Flow Cytometry

Plots

Confirm that:

☒ The axis labels state the marker and fluorochrome used (e.g. CD4-FITC).

☒ The axis scales are clearly visible. Include numbers along axes only for bottom left plot of group (a 'group' is an analysis of identical markers).

☒ All plots are contour plots with outliers or pseudocolor plots.

☒ A numerical value for number of cells or percentage (with statistics) is provided.

Methodology

Sample preparation	Single-cell suspensions from lungs were obtained by collagenase digestion. Cells were stained with fluorochrome-conjugated antibodies, according to the manufacturer’s protocols. Flow cytometry was performed with a BD FACSFertasa flow cytometry system (BD Biosciences, San Jose, Calif), and data were analyzed with FlowJo software (Tree Star, Ashland, Ore).
Instrument	FACSVerse and FACSAria II (BD Biosciences)
Software	Flow cytometry data was analyzed by Flowjo v10.<br>Data analysis and plotted were using GraphPad Prism 8.<br>BD DIVA 8.0.1 sofewate (BD Biosciences).
Cell population abundance	The abundance is depended on the specific population, from 5%-80%
Gating strategy	All the gating strategy for each experiment was supplied in supplementary figures. They were determined by single color stains where applicable. Generally, the first gating (FSC/SSC) was done on alive cells to exclude debris and dead cells, followed by the section of single cells (FSC-W/FSC-H), excluding clumps. Then the specific fluorophores were confirmed and analyzed by comparing single color stains.

☒ Tick this box to confirm that a figure exemplifying the gating strategy is provided in the Supplementary Information.
